# Recent Updates on Research Models and Tools to Study Virus–Host Interactions at the Placenta

**DOI:** 10.3390/v12010005

**Published:** 2019-12-18

**Authors:** Jae Kyung Lee, Soo-Jin Oh, Hosun Park, Ok Sarah Shin

**Affiliations:** 1 Department of Biomedical Sciences, College of Medicine, Korea University Guro Hospital, Seoul 08308 Korea; jae.lee0321@gmail.com (J.K.L.); sjooooh@gmail.com (S.-J.O.); 2 Department of Microbiology, College of Medicine, Yeungnam University, 170 Hyeonchung-ro, Namgu, Daegu 42415, Korea

**Keywords:** placenta, congenital infection, immunity, trophoblasts, Hofbauer cells

## Abstract

The placenta is a unique mixed organ, composed of both maternal and fetal tissues, that is formed only during pregnancy and serves as the key physiological and immunological barrier preventing maternal–fetal transmission of pathogens. Several viruses can circumvent this physical barrier and enter the fetal compartment, resulting in miscarriage, preterm birth, and birth defects, including microcephaly. The mechanisms underlying viral strategies to evade the protective role of placenta are poorly understood. Here, we reviewed the role of trophoblasts and Hofbauer cells in the placenta and have highlighted characteristics of vertical and perinatal infections caused by a wide range of viruses. Moreover, we explored current progress and future opportunities in cellular targets, pathogenesis, and underlying biological mechanisms of congenital viral infections, as well as novel research models and tools to study the placenta.

## 1. Introduction

As a highly specialized organ present only during mammalian pregnancy, the placenta creates an environment that is suitable for fetal growth and development through tight regulation and coordination. The placenta, which is located at the interface between the mother and the fetus, takes on a broad range of functions, such as respiration, excretion, and protection, and gives rise to different cell types during the developmental process to carry out its duties. The placenta is comprised of both maternal and fetal tissues, which are derived from the endometrium and chorionic sac, respectively, and the intervillous space located between the two regions contains placental villi that are essential for maternal–fetal exchange [1,2]. Trophoblasts are the precursor cells that lead to human placental cells and originate from the outer layer of the blastocyst, which is a structure that forms after the embryo is fertilized and develops into the placenta surrounding the fetus contained inside [3]. Thus, trophoblasts, among many cellular components of the placenta, are mainly responsible for placental structure and functionality. Since the placenta regulates the supply of nutrients directed towards the growing embryo, malfunctions in the placenta can result in complications such as miscarriage, stillbirth, preterm birth, and abnormalities of fetal growth and development including fetal growth restriction (FGR) and microcephaly [4]. Furthermore, pregnancy-related complications are often caused by a combination of multiple factors involved in placental development. Therefore, greater understanding of the placenta, along with its implications in fetal development, especially on a cellular and molecular level, is necessary to address these complications.

In addition to the exchange of nutrients, the placenta is also a designated site for the transfer of various hormones, drugs, and pathogens. As a result, the placenta also takes on a protective role against pathogens that may cross the maternal–fetal barrier. Recent studies have confirmed the vertical transmission of Zika virus (ZIKV), during which maternal infection with the virus results in pregnancy-related complications and congenital abnormalities, including miscarriage and microcephaly [5,6,7]. These findings, in turn, highlight the ability of viruses like ZIKV to develop strategies to invade and target placental cells for infection, in addition to demonstrating the importance of placental immunity in normal fetal growth and development. Unfortunately, the exact mechanisms employed by certain viruses for immune evasion at the placenta have not been identified, and further studies are necessary to clarify viral strategies to circumvent placental defense mechanisms. Moreover, the experimental challenges associated with studying the human placenta using conventional models can be gradually overcome using novel platforms that rely on microfluidic and microfabrication techniques [8]. This approach has enabled the placenta to be recreated and studied in terms of its function as a barrier at the maternal–fetal interface.

In this review, we have examined cellular components of the placenta and provide insight into placental immunity and its importance in successful fetal growth and development. In addition, the review highlights current progress and future opportunities associated with studying placental pathogenesis, and introduce novel experimental platforms that can be used to study the biological mechanisms of viral congenital infections.

## 2. Cellular Components of the Placenta 

**Figure 1:**
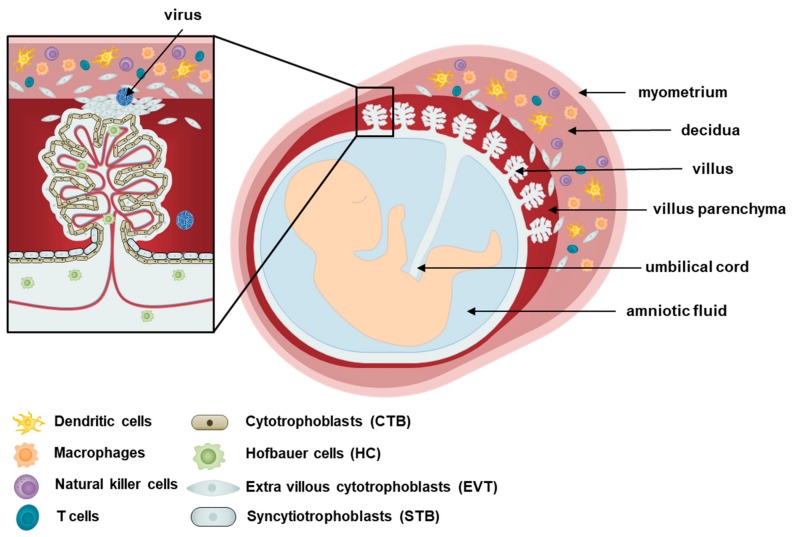
Cells of the placenta. A schematic diagram of the human placenta during pregnancy is shown. The human placenta contains three main types of epithelial trophoblasts: cytotrophoblasts (CTBs), syncytiotrophoblasts (STBs), and extravillous trophoblasts (EVTs). The CTBs are mononuclear cells at the fetal interface that eventually differentiate via cell-to-cell fusion into STBs. The STB layer is a multinucleated structure that covers the entire surface of the villous tree throughout pregnancy that is bathed in maternal blood, and mediate nutrient and gas exchange between mother and fetus. Hofbauer cells (HC), macrophages of fetal origin, are found in the intervillous spaces, while EVTs migrates from the chorionic villi, invades into the uterine wall, and remodels maternal spiral arteries to facilitate blood supply of the placental unit. In addition to the EVTs, the decidual compartment also includes maternal immune cells (eg, decidual dendritic cells, macrophages, natural killer cells and T cells) and stromal cells. EVT, extravillous cytotrophoblasts; CTB, cytotrophoblasts; HC, Hofbauer cells; STB, syncytiotrophoblasts.

The human placenta is a disc-like structure attached to the uterine wall (decidua) and connected to the fetus via the umbilical cord. Placentation, the formation of the placenta, begins as trophoblasts of the fetal blastocyst origin interact with the decidua basalis of maternal endometrium origin. The placenta develops diverse villi containing different types of specialized cells, as shown in Figure 1. The placenta is assembled by the chorionic villi, which comprise floating villi and anchoring villi. The floating villi are mainly responsible for transporting waste, nutrients, and gases between the mother and the fetus, while the anchoring villi support the decidua [9]. Various subsets of maternal immune cells constitute the decidual immune system, such as macrophages, dendritic cells, natural killer cells, and T cells [10].

At the outermost layer of the placenta, two populations of trophoblasts are responsible for coordinating maternal–fetal interactions, and serving as an initial line of defense against pathogens [11]. Syncytiotrophoblasts (STBs) are a multinucleated, fully differentiated population of trophoblasts that form a continuous layer above cytotrophoblasts (CTBs), which are undifferentiated mononuclear stem cells of the placenta that are able to differentiate into other trophoblast types [12]. While CTBs form the anchoring villi, STBs form the floating villi and are responsible for the exchange that occurs at the villus surface between maternal blood and fetus [13]. Aggregates of CTBs also organize into extravillous cytotrophoblasts (EVTs), which are placental trophoblasts that invade the maternal decidua, anchor the placenta, and subsequently lead to the transfer of nutrients to the fetus [9,14]. In addition to separating the fetus from maternal tissues, the layer composed of STBs and CTBs is particularly important in terms of protecting the semi-allogenic fetus against maternal immune attacks [15]. STBs and CTBs do not express MHC class I and II molecules, and this lack of expression may explain why the maternal immune response tolerates the fetus [16,17]. The expression of MHC class I molecules by EVTs has been suggested to aid in the invasion of the maternal decidua. It is important to note that abnormalities in the EVT-induced invasion of the maternal blood vessels are associated with pregnancy complications such as FGR and even pregnancy loss [3]. Trophoblasts are also known to secrete high levels of extracellular vesicles (EVs) enriched in non-coding RNAs, such as microRNAs (miRNAs) [18]. These EVs are involved in maternal–fetal communication and possibly antiviral activity. As an example, isolation and characterization of EVs from human trophoblasts in Ouyang et al. demonstrated the antiviral activity of placental EVs derived from human trophoblasts [19]. These findings emphasize the important immunoregulatory role of placental trophoblasts.

The placenta also contains an extensive number of mesenchymal stem cells (MSCs) of fetal origin, including amnion mesenchymal stromal cells, chorionic villi mesenchymal stromal cells, and decidua mesenchymal stromal cells, which can differentiate into endothelial cells or macrophages known as Hofbauer cells (HCs). Analysis of HCs in Reyes et al. has revealed that these cells are heterogeneous, and the diversity of HCs differs throughout pregnancy, during which the first and third trimesters gave rise to more diverse populations [20]. As antigen-presenting cells of the placenta, HCs, in conjunction with trophoblasts, are mainly responsible for protecting the fetus from pathogens or toxins, and have been shown to adopt an M2 polarity phenotype as opposed to M1, which is characteristic of alternatively activated macrophages [20,21]. While M1 macrophages are able to kill microbes, M2 macrophages are associated with regulating and inhibiting inflammatory and immune responses as part of the repair machinery following an infection [22]. M2 polarization, therefore, enable HCs to serve as possible reservoirs of pathogens in the placenta, contributing to the vertical transmission of pathogens from the mother to the fetus. In addition to placental immunity, HCs are also important regulators of trophoblast differentiation, vasculogenesis, and angiogenesis through their production of cytokines and growth factors [23,24]. These placental macrophages are able to fulfil a role in placental development, fetal protection, and intercellular communication through their ability to be motile, phagocytose exogenous antibodies and to associate in groups, as demonstrated by in vitro and in vivo studies using various experimental techniques, such as microscopy, flow cytometry, and immunohistochemistry [25,26].

While different types of cells emerge throughout the stages of gestation, placental cells have demonstrated immunomodulatory characteristics and expression of markers associated with stem cells, both of which have made the placenta an important source of cells with potential regenerative and reparative properties [27,28,29,30]. Igura et al. suggests the placenta as a viable source of MSCs and, upon stimulation, these placental MSCs from the fetal chorionic villi possess the ability to differentiate into the following mesenchymal lineages: adipocytes, osteocytes, and chondrocytes [31]. Characterization of placental MSCs using real-time polymerase chain reaction (PCR) and flow cytometry has revealed that they possess stem-cell-like gene and protein expression profiles, in addition to their immunomodulatory and migratory properties [32]. Luan et al. analyzed the immunosuppressive properties of placental MSCs using ELISA, the results of which showed that placental MSCs inhibit the proliferation and interferon (IFN)-γ secretion of T cells [33]. Although placental stem cells have exhibited limited plasticity in most studies due to the experimental challenges associated with studying the placenta, the differential potential of these cells highlights the placenta as a promising therapeutic tool with advantages over conventional methods for obtaining stem and progenitor cells, such as using bone marrow harvested from donors.

## 3. Viral Infections during Pregnancy

The placenta is considered a site of immune privilege, in the sense that immune responses to pathogens are downregulated to a certain degree to ensure proper function of the placenta [34]. Therefore, the immune response and tolerance must be balanced at the maternal–fetal interface to allow the exchange of nutrients and waste while inhibiting harmful pathogens from crossing the placenta. However, the immunotolerant environment created during pregnancy may permit viral transmission, with the placenta serving as a portal for viral entry. Maternal infections can induce an inflammatory process that poses a major threat to the developing fetus and results in pregnancy complications ranging from miscarriage to preterm birth, malformation, and intrauterine fetal demise [35]. Table 1 shows some of the viruses that can cause vertical and perinatal infections. Some of the most common pathogens that cross the placenta to infect the fetus are collectively referred to as “TORCH,” which stands for *Toxoplasma gondii*, others (including varicella zoster virus), rubella, cytomegalovirus (CMV), and herpes simplex virus (HSV) [36]. Based upon its effect on the fetus and development of the congenital Zika syndrome, Zika virus has emerged as the newest TORCH agent [37].

Rubella virus (RV), belonging to the *Togaviridae*, can cause congenital abnormalities which have increasing severity when the maternal infection occurs during the first trimester of pregnancy. While RV infections normally result in mild symptoms in both children and adults, congenital infections manifest as various birth defects, including microcephaly, that are collectively known as congenital rubella syndrome (CRS), which damages the fetal heart and blood vessels [38]. Although the transmission route taken by RV to the placenta has yet to be studied, viral antigens have been detected in the chorionic villi, within CTBs and endothelial cells, and viral replication in the fetal endothelial cells results in a different transcriptional profile in comparison to adult cells that were infected with RV in vitro [39,40]. The change in gene expression profiles of the infected fetal cells may be explained by the downregulation of genes involved in cytokine production and regulation, in addition to fetal development of sensory organs. RV-induced apoptosis has also been suggested as a contributing factor to CRS, as evident from the cellular damage in sites, such as the eyes, heart, brain, and ears [41,42,43]. Despite the detrimental pathologies associated with RV infection in the fetus, further studies are necessary to understand RV-mediated invasion of the fetal compartments.

As a member of *Herpesviridae* family, herpes simplex virus (HSV) is also associated with the risk of fetal transmission that can lead to abnormalities, especially in the central nervous system of newborns. The majority of HSV infections during pregnancy are caused by HSV-2, and there exists greater risk of HSV transmission during vaginal birth [44]. HSV infection during pregnancy can result in miscarriage and also FGR, neurological complications including microcephaly, and even stillbirth. In addition, another member of the *α−herpesviridae* Varicella zoster virus (VZV) may cause congenital varicella syndrome (CVS) during the first two trimesters of pregnancy. Although rare, congenital VZV infections can result in serious fetal manifestations, including neurodevelopmental defects when the infection occurs during the first two trimesters [45]. Cellular and molecular analysis has revealed that VZV DNA can be detected in the placenta and amniotic fluid, while VZV is able to successfully replicate in CTBs [46]. Although the exact mechanisms of VZV transmission in the placenta are unknown, VZV-infected T cells can be localized to the basal decidua, where VZV replicates and spreads to the adjacent placenta in the intervillous blood space [13].

Human cytomegalovirus (CMV) also belongs to the herpes virus family that includes HSV-1, HSV-2, and VZV [47]. Congenital infection with CMV is of great public health significance due to the wide range of birth defects the virus can cause, including FGR and neurological complications like microcephaly. Interestingly, the clinical spectrum of congenital CMV infections has been found to be variable, with presentations ranging from asymptomatic infection to potentially life-threatening disseminated disease. Despite its clinical significance, congenital CMV infection is often not diagnosed properly because the majority of infected infants are asymptomatic at birth and screening programs have not been substantially implemented [48]. Although the virus can be transmitted from the mother to the fetus throughout the duration of pregnancy, maternal infection during the first trimester causes the most severe disease in infants [49,50,51]. Analysis of the interaction between CMV and placenta using immunochemistry has shown that CMV viral proteins are expressed in CTBs, fibroblasts, macrophages, and STBs, among which CTBs were particularly permissive for CMV replication [52].

Group B Coxsackievirus (CVB) is a *Picornaviridae* enterovirus, with which infection during pregnancy causes serious and sometimes fatal outcomes for the fetus [53,54]. Infections with Coxsackievirus B3 (CVB3) during late pregnancy and delivery have been reported to have significant effects on the fetus, including neurological defects, encephalitis, myocarditis, meningitis, and even death [55,56,57]. According to Hwang et al., when cases of early pregnancy loss were analyzed for the prevalence of enterovirus infection using RT-PCR and immunohistochemistry, the rate of CVB3 infection in cases of abortion was 57.1% [58]. Despite the high rate of fetal death associated with CVB infections, there are limited data regarding the outcome of infection during early pregnancy, since early maternal infections are commonly asymptomatic and therefore undetected. Coxsackievirus and adenovirus receptor (CAR) expression at the host cell surface is essential for viral entry and internalization of CVB3, and the receptor is not only highly expressed in the fetal brain, but also a crucial factor in embryonic development of the heart [59,60]. Hwang et al. investigated the outcomes of early CVB3 infection during pregnancy in ICR mice, demonstrating the vertical transmission of CVB3 enabled by the high expression level of CAR in the uterus and embryo of the pregnant mice [61]. CVB3 replication, as analyzed by RT-PCR and plaque assays, was confirmed in the embryos and placentas of the CVB3-infected mice, whose embryos were particularly fragile in the brains and hearts [61]. Furthermore, Euscher et al. showed localization of CVB RNA and protein in HCs, STBs, and CTBs of human placental tissue harvested from newborn infants [62].

Clinical observation of birth defects during the recent epidemic has also emphasized ZIKV as an important threat to public health in the Americas. ZIKV is a mosquito-borne flavivirus that can undergo vertical transmission to cause serious fetal defects, including microcephaly and abnormal central nervous system development [63,64]. Previous reports have confirmed ZIKV RNA in the human fetal brain and amniotic fluid [65,66]. Accumulating evidence suggests that cells in the placenta are major targets of ZIKV. In particular, Bayer et al. demonstrated that primary human placental trophoblast cell lines are permissive to ZIKV infection, whereas Schwartz, Rosenberg, and Quicke et al. confirmed the presence of ZIKV infection in HCs, in addition to hyperplasia of HCs and the lack of an inflammatory response in the placenta in response to the infection [67,68,69,70]. Agaard et al. suggested that the receptors necessary to mediate ZIKV entry are expressed during the differentiation process of placental trophoblasts to CTBs and STBs, and allow ZIKV replication to occur in the trophoblasts [71]. These observations highlight the potential role of the placenta as a reservoir and entry for the virus to reach the fetus and target the trophoblast and HC populations. Because microcephaly is most likely caused by an abnormal development of fetal neural stem cells (NSCs), McGrath et al. also investigated the effects of ZIKV infection on the neuronal differentiation of human NSCs (hNSCs) [72,73]. Transcriptomic analysis revealed that certain strains of ZIKV induce alterations in gene expression of hNSCs, such as upregulation of genes functional in the innate immune response, inflammation, and apoptosis, and downregulation of pathways involved in the cell cycle and neural development. Also a member of the *Flaviviridae* family, Dengue virus (DENV) can also infect the fetus through vertical transmission and result in complications for both the mother and fetus, including maternal death, miscarriage, and stillbirth [74].

In addition to ZIKV, Ebola virus has also caused recent outbreaks that caused a public health emergency. Ebola virus infection during pregnancy threatens the fetus: in nearly all cases, Ebola infection in pregnant women has resulted in miscarriage, stillbirth, or neonatal death [75]. Given that Ebola virus research has to be done in a biosafety laboratory level 4 facility, the pathophysiology or maternal fetal transmission mechanisms are yet to be identified. Meanwhile, studies involving immune correlates of protection or transmission at the placenta for human immunodeficiency virus (HIV) have been somewhat established. It is known that HCs are key mediators of HIV transmission, limit HIV-1 replication, and potentially offset mother to child transmission by induction of immunoregulatory cytokines [76]. Furthermore, many host restriction factors expressed by HCs or trophoblasts have been identified, including APOBEC3G and ISGs [77,78]. Despite this, the detailed mechanisms underlying HIV’s strategies to evade the protective role of placenta are poorly understood. In to the same Filoviridae family, Marburg virus can also infect the placenta and result in similar complications for the fetus as Ebola virus [79]. Lassa virus is a member of the *Arenaviridae* family, and an infection with Lassa virus manifests as similar symptoms to an infection with Ebola virus, due to the similar cellular targets that are shared between the two viruses [80]. Lassa fever is a hemorrhagic disease caused by Lassa virus, and the increasing occurrence of this disease has highlighted Lassa virus as an important pathogen that can lead to 90% perinatal mortality when infection occurs during pregnancy [81].

Other viruses that are capable of establishing a placental infection include parvoviruses. Vertical transmission of parvoviruses can be explained by parvovirus B19 receptor expression by placental trophoblasts and erythroid precursor cells [82]. B19 infection during pregnancy can result in complications such as spontaneous abortion and intrauterine fetal death, and Pasquinelli et al. has demonstrated that placental endothelial cells can also be infected with parvovirus B19 [83,84].

Current laboratory methods for determining viral infections during pregnancy rely on serology, which measures levels of IgM and IgG, and virus detection, including virus isolation, and molecular assays for the detection of viral nucleic acid [85]. However, greater efforts are needed to help pregnant women and newborns to avoid the risk of infections and their consequences. The ability of the previously mentioned pathogens to cross the placental barrier and ultimately infect the developing fetus highlights the need to identify the mechanism underlying maternal–fetal transmission. Findings from previous studies emphasize the important role of fetal placental cells as possible reservoirs and conduit for pathogens at the maternal–fetal interface. Unfortunately, the exact mechanisms underlying viral infections in the placenta remain to be identified. Further studies on vertical transmission of pathogens, and investigation of the interactions between the viruses and the host at the placenta, in particular, are crucial for the discovery and development of vaccines and therapies to prevent and treat congenital infections.

## 4. Research Models to Investigate Placental Pathogenesis

**Figure 2:**
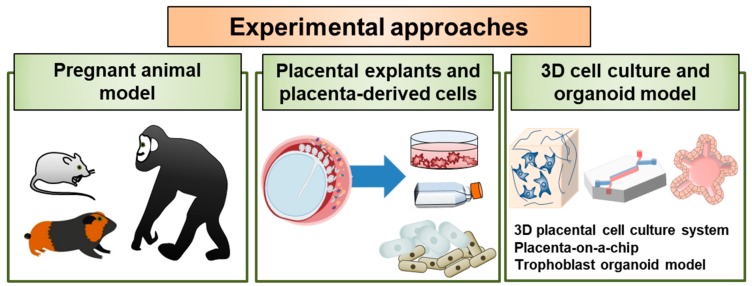
Experimental approaches to study host–pathogen interactions at the placenta. The utility of currently described or potential models of viral infections in the placenta is illustrated. Research models are employed for the investigation of basic features of viral infection and pathogenesis and to investigate unusual features of viral pathogenesis, including sexual transmission, transplacental transmission, and congenital malformations in developing fetuses. In addition, these models could be used in the future to evaluate candidate vaccines and therapeutics for the prevention and treatment of viral disease in individuals and in fetuses during infection of pregnant hosts.

The study of early human placental development is hampered by practical and ethical issues, and both animal and in vitro cell culture models are routinely used to study the essential functions of the placenta. In particular, choriocarcinoma-derived cell lines such as BeWo, Jar, and Jeg-3, can be alternatives for and complementary to the primary cell models as in vitro models for placental research. Given the significant spatiotemporal differences in early placental development in rodents and humans, it is important to develop new experimental approaches to study host–pathogen interactions at the maternal–fetal interface during pregnancy. It is also critical for us to elucidate the pathways and defense mechanisms associated with viral infection during pregnancy and further develop effective and safe vaccines. In the following sections, we have summarized current experimental models that can be used to study placental pathogenesis and discuss emerging opportunities to enhance our understanding of the crosstalk between the maternal and fetal compartments (Figure 2).

### 4.1. Pregnant Animal Model

Due to the unique characteristics of human placentation, an ideal model to study human pregnancy remains to be established. However, animal models have proven to be valuable research tools in understanding placental development and function, and range from non-human primates, guinea pigs, and mice to horses and sheep [86]. Small animal models, including mice, rodents, and guinea pigs, have a short gestational period and give birth to poorly developed young, whereas larger animals, such as non-human primates, which have longer gestational period, comparable to that of humans, and give birth to young with relative maturity [87]. The morphological and functional diversity of the placenta between different species have allowed different aspects of human pregnancy to be studied in suitable animal models. Table 2 outlines similarities and differences between humans and animal models frequently used in the study of placental development and function.

Mice have been the most frequently used animal models not for their similarities to humans, but for the practicality of their small size and short generation times. Mice and humans share similar placental cell types and genes that regulate placental development, but trophoblast invasion in mice is shallow and limited relative to the extensive invasion of maternal uterine vessels that occurs during human pregnancy [88,89]. In both mouse and human placenta, STBs cover the villi and are in direct contact with the maternal blood [90]. Mouse mutants, specifically, have been useful in understanding the different genetic pathways involved in controlling placental development, and how morphogenesis and placental cell differentiation can be affected in these mutants. Furthermore, studies of genes associated with placental defects in mice have demonstrated the importance of placental defects as a major factor contributing to abnormal embryonic development [91]. According to Perez-Garcia et al., co-occurrence of placental defects and embryonic defects are more common than previously thought, and are often accompanied by defects in the fetal heart and brain, as observed in mouse models.

Guinea pigs are often selected as an animal model to study fetal growth restriction and transfer of substrates across the placenta. Jansson and Persson et al. demonstrated using guinea pigs that growth restriction is linked to an impaired placental transfer, resulting in a reduction of the amino acids available [92]. In addition, Dyson et al. underlined guinea pigs as a suitable model for the study microvascular dysfunction in relation to preterm birth and neonatal mortality [93]. Guinea pigs have also been used extensively as an experimental model to study congenital CMV infections [94,95,96]. CMV replication has been observed in trophoblastic cells of the guinea pig, demonstrating the ability of guinea pigs to model human CMV infections at the placental level, and the occurrence of infection in the fetus [97]. Similarly, guinea pigs have been used to model the placental pathology of ZIKV. Immunocompetent guinea pigs are susceptible to infection by a contemporary strain of ZIKV [98,99].

The similarities of trophoblast cell types between horses and humans have made horses another animal model used in the study of pregnancy immunology. Although the equine placenta is classified as diffuse, with maternal–fetal exchange occurring across all available surface, the trophoblast populations of human and horse placentas share significant phenotypic similarities. Non-human primates could be considered the best animal model to study placental conditions due to their important similarities to humans. However, their use in biomedical research is greatly limited due to important ethical questions, as well as their high cost. Apes resemble humans in having interstitial implantation, while chimpanzee and gorilla resemble humans in the routes and extent of trophoblast invasion during placentation.

### 4.2. Placental Explants and Placenta-Derived Primary Cell Models

The maternal–fetal interface has been studied using cultures of human placental explants, a method that requires optimization to mimic the in utero environments of gestational periods through differential pressure, culture medium, and extracellular matrices. Viral pathogenesis has also been modeled in placental and decidual tissue explants, demonstrating which placental cell types are susceptible to infections and providing insight into specific transmission routes in the placenta [112]. First-trimester human placental explant culture by Genbacev et al. was used to show that cytotrophoblasts are vulnerable to infection with ZIKV strain MR766, whereas cytotrophoblasts cultured from full-term placental explants in Bayer et al. were not infected by MR766 due to the high level of type III interferon secretion [68]. In Platt et al., neurotropic flaviviruses related to ZIKV, including West Nile virus, were shown to be able not only to cause placental infection, but also to replicate efficiently in second trimester placental explants of the decidua and chorionic villi and fetal membrane [113]. These studies highlight the importance of placental explants as culture models to study placental pathogenesis and identify the pathogens that are capable of vertical transmission causing cause fetal infection and injury.

Although animal models provide useful platforms for supplementing and elevating cell-based in vivo studies, these techniques fail to reflect the physiological parameters and cellular communication occurring in human conditions, which are essential to understanding the mechanisms of placental development and disease. Given the limitations of the pregnant animal model, it will be ideal to characterize virus–host interactions using primary cells isolated from human placenta. Single-cell RNA and DNA sequencing can help to identify the characteristics of maternal and fetal cells in the decidua and placenta, and how these cells interact with one another. Recently, a comprehensive single-cell transcriptomics atlas of the maternal–fetal interface during early pregnancy was completed by Vento-Tormo et al. [114]. Researchers mapped over 70,000 single cells at the junction of the uterus and placenta, revealing how cells cross-talk to each other to modulate immune response and maintain pregnancy. Furthermore, single-cell RNA sequencing has revealed the diversity of trophoblast subtypes and patterns of differentiation in the human placenta. Different cell subtypes from placenta secrete diverse polypeptide hormones, as a source of many hormones involved in fetal growth and maternal adaptation to pregnancy. Newly identified cell types have been reported, including CD68-positive HBCs called macro_1 and macro_2 [115].

### 4.3. 3D Cell Culture and Organoid Models

To address the limitations of conventional two dimensional cell culture techniques, researchers in stem cell and developmental biology have been making efforts with engineers and physical scientists to develop advanced in vitro technologies for three dimensional (3D) cell culture models. Reconstructions of host microenvironments using 3D tissue culture, multicellular complexity, microbiota composition, and biomechanical forces allow researchers to mimic essential features present in the native host microenvironment. This recent shift to studies utilizing 3D cell culture models has given rise to the following three models: 3D cell cultures engineered in the rotating wall vessel (RWV) bioreactor, extracellular-matrix-embedded/organoid models, and organ-on-a-chip models [116].

RWV is the most commonly used platform for suspension culture, providing cells with the spatial freedom to display their natural affinities to co-localize and self-assemble, and this model is particularly useful for studying the innate immune response to microbial infections [117]. As an example, McConkey et al. recently described how that the trophoblast JEG-3 choriocarcinoma cell line recapitulates the morphological and secretory phenotypes associated with primary syncytiotrophoblasts when co-cultured in 3D with microvascular endothelial cells [118]. Additionally, 3D organoid models can be developed from stem cells to mimic in vivo tissues, and Turco et al. generated human trophoblast organoids that can be used to study development and dysfunction at the placenta, in addition to being viable for long-term culture [119]. Nanotechnology has also enabled organs to be constructed on a microdevice, known as the organ-on-a-chip. An organ-on-a-chip can be broadly defined as a microfabricated cell culture device designed to model the functional units of human organs in vitro [120]. Several groups have reported the development of placenta-on-a-chip microdevices for the study of complex placenta responses. The placental barrier can be simulated in vivo to create a 3D microenvironment and flow system in both trophoblast and endothelial cells, resembling the dynamic environment in maternal and fetal circulations in the body [8,119,120,121,122]. Yin et al. modeled the placenta barrier using the organ-on-a-chip technology to investigate transfer at the maternal–fetal interface by co-culturing placental cells to mimic the placenta in vivo [122].

Although these research models have various limitations in terms of modeling specific interactions at the placenta, these platforms still allow physiological conditions of the host to be recreated for interface of host–pathogen interactions. Exploring how these recently developed technologies may be leveraged to address the major technical challenges in studying the placenta will lead to greater understanding of this highly specialized organ.

## 5. Current Progress and Future Perspectives of Studying Maternal–Fetal Interface

Using the research models mentioned above, the influence of different immune signaling pathways following viral infections during pregnancy, particularly those of the placenta and fetal development, could be investigated and answer the following long-standing questions. Why do some maternal infections, but not others, lead to congenital diseases? What are the cellular sources and targets of virus in the placenta and the fetus? What are the molecular mechanisms of virus-induced host damage in target cells?

Perhaps we can learn from remarkable progress in ZIKV research, which has provided significant insight into the role of immune cells, cytokines, and viral virulence during pregnancy in the past few years. For examples, Foo et al. identified CD14^+^ monocytes as the primary target for both African- and Asian-lineage ZIKV infection from pregnant women [123]. Interestingly, there was differential immunomodulatory response in the monocytes of these pregnant women, in that African-lineage ZIKV infection led to M1-skewed inflammation, whereas Asian-lineage ZIKV infection led to M2-skewed immunosuppression.

Distinct roles of type I vs. type III IFNs were also highlighted in ZIKV research. Although the deficiency of IFN-αR in mice can lead to higher ZIKV titers in the placentas of offspring ZIKV titers, while exposure of midgestation human chorionic villous explants to type I, but not type III IFNs alters placental morphology, resulting in abnormal architecture of the maternal–fetal barrier [124,125]. On the other hand, type III IFNs have been suggested to play an important role in host defense. Human trophoblasts constitutively release type III IFNs, which function in both a paracrine and autocrine manner to protect trophoblast and non-trophoblast cells from ZIKV infection [68,126]. A recent report by Caine et al. indicates the defensive role of type III IFNs in the female reproductive tract [126]. In particular, mice lacking IFN-λ signaling sustain greater female reproductive tract infection when progesterone is administered. Exogenous IFN-λ treatment confers an antiviral effect when mice receive both estradiol and progesterone, but not progesterone alone. Further studies will be necessary to delineate the pathways by which ZIKV accesses the fetal compartment, evades restriction by trophoblast-derived IFNλ1 and other trophoblast-specific antiviral factors, and/or uses alternative strategies to cross the placental barrier.

Current progress in understanding the molecular defense mechanisms of placental cells has been focused on components secreted by trophoblasts: miRNAs and exosomes. Analysis of the miRNA expression profiles in the placenta has revealed that a cluster of placenta-specific miRNAs linked to chromosome 19, in particular, are differentially expressed by villous trophoblasts during placental development [127,128]. Among many miRNAs expressed by human trophoblasts, the chromosome 19 microRNA cluster (C19MC) constitutes the largest human miRNA cluster, and C19MC miRNAs are expressed from chromosome 19q13.41 [129]. This cluster includes 58 mature miRNA species that are encoded by 46 miRNA genes, spanning over 100 kb. Intriguingly, this cluster is expressed exclusively in primates, and expressed almost exclusively in the placenta [18,128,130,131]. Study of this miRNA secretion using the BeWo cell line revealed that STBs were the main source of the miRNAs released into the maternal circulation, and the differential expression of C19MC miRNAs regulates placental physiology [127].

Placental trophoblasts are known to produce high levels of exosomes [18]. Extracellular miRNAs can be initially packaged and secreted by exosomes, which are small EVs derived from placental trophoblasts, mainly involved in intercellular communication [132,133,134]. Being enclosed in exosomes might explain why miRNAs circulating in plasma are highly stable. Exosome-mediated transfer of miRNAs specific to the placenta is one mechanism of maternal–fetal communication, especially in the context of regulating maternal immune response to pathogen infections. Valadi et al. have demonstrated the ability of exosomes to shuttle various RNA species that can be translated and ultimately regulate the activity or differentiation of these recipient cells [132]. Moreover, exogenous expression of the entire C19MC in non-placental cells via exosome-mediated delivery drastically reduces virus infection, and select expression of C19MC family members miR517-3p, miR516b-5p, and miR512-3p alone significantly limited the replication of both RNA and DNA viruses [18,135]. Together, these findings demonstrate the importance of placental trophoblasts at the maternal–fetal interface in terms of limiting the spread of pathogens through miRNA-mediated regulation.

Going forward, investigation of how the microbiome is associated with placental pathology is a new area of interest. Increasing evidence indicates a link between preterm birth and the microbiomes of tissues previously thought to be sterile, including the placenta. Among the first evidence of a placental microbiome is the finding by Aaggard et al. that bacteria are found in the placenta in full-term pregnancies in the absence of histological inflammation and clinical infection [136]. Furthermore, the placental membrane microbiome is altered among subjects with spontaneous preterm birth with and without chorioamnionitis [137,138,139,140]. As expected, pregnancies that resulted in spontaneous preterm births were associated with placental microbiota that varied depending on the severity of chorioamnionitis, in addition to significant alterations in certain bacterial metabolic pathways, which may be contributing factors to an increased risk of preterm birth. Ferretti et al. have also suggested vertical transmission of microbes from multiple maternal sites of the body, among which the mouth and gut make the greatest contributions to microbial diversity [141]. In contrast, a recent study reported that bacterial infections of the placenta are uncommon and do not account for the majority of cases of pregnancy-related complications, including preterm birth [142]. According to de Goffau et al., bacterial acquisition by infants occurs during labor and delivery, and a healthy human placenta does not contain a microbiome. However, one cannot rule out vertical transmission of microbes and, given the possibility that placental membrane microbiome altered by viral infection may also contribute to the risk of miscarriage, preterm birth, and microcephaly, metagenomic sequencing studies will be very important to identify bacterial species unique to the placenta and alteration patterns in the microbiome composition.

Lastly, the interactions of hormones and the immune system contributing to both the outcome of pregnancy and female susceptibility to viral infections should be further studied. Female reproduction is regulated predominately by estrogen, progesterone, luteinizing hormone, and follicular stimulating hormone. These sex hormones contribute significantly to the shift in immune function that occurs over the three trimesters of pregnancy. Pregnant women have a unique immunological profile modulated by the sex hormones required to maintain pregnancy, namely progesterone and estrogens. Insufficient progesterone production has been associated with infertility and recurrent miscarriages [143,144], whereas estrogens have also been implicated in inducing CD4^+^ CD25^+^ T regulatory cells (T_regs_) and are critical for maintaining tolerance within the maternal–fetal interface [145]. Progesterone also upregulates the activity of uterine T_regs_, which act as suppressors of inflammatory immune subsets, particularly NK cells and macrophages resident to the endometrium [146]. Thus, hormonal regulation of pregnancy and immune signaling are delicately balanced to protect fetal development. However, this balance can be disrupted by viral pathogens that cross placental barrier. For example, Littauer and colleagues demonstrated that influenza virus infection disrupts progesterone production and upregulates inflammatory mediators such as cyclooxygenase-2 and prostaglandins, resulting in preterm birth and miscarriages [147]. Furthermore, using an animal model, the same group reported that viral load was negatively associated with progesterone concentration, and reduced progesterone expression was correlated with preterm birth in influenza virus-infected pregnant mice [148]. Administration of progesterone to female mice following influenza A (H1N1) virus infection reduced immunopathological changes and improved lung epithelial cell regeneration, although it did not reduce viral load [149].

## 6. Conclusions

The epidemics of ZIKV and its clinical consequences during pregnancy raised awareness of the importance of research into placental pathology and immunology. Although many risk factors have been taken into consideration in identifying the etiological agents of miscarriage, infertility and birth defects, many cases of pregnancy complications are yet to be accompanied by a clear explanation. As we previously summarized, certain viral infections can lead to detrimental defects on the developing fetus. Several studies have confirmed the role of viral infections as a direct cause of miscarriage and intrauterine fetal demise, especially during the second trimester of pregnancy. However, the association between infection during the first trimester and miscarriages is still questionable. Therefore, it will be important to further characterize cellular and molecular virulence mechanisms of viral pathogenesis using cells or tissues derived from the placenta harvested from first trimester pregnancies.

Once thought to provide a passive structural and physiological barrier at the maternal–fetal interface, the placenta is now emerging as an important regulator of immune response during pregnancy. Future modeling of viral infections in the placenta using novel platforms such as 3D cell cultures or organ-on-a-chip devices will address the extent of pathophysiology associated with viral infections and the underlying biological mechanisms that can lead to the development of therapeutics to prevent pregnancy complications.

## Figures and Tables

**Table 1:** Viruses that infect the placenta via vertical or perinatal route. FGR: fetal growth restriction.

Virus	Family	Host	Typical transmission route	Pregnancy outcomes
**Rubella virus**	*Togaviridae*	Humans	Aerosols, secretions	Miscarriage, cognenital rubella syndromes (hearing loss, cataract, congenital heart disease, microcephaly etc.)
**Herpes simplex virus**	*α-herpesviridae*	Humans	Oral or sexual contact	Miscarriage, FGR, stillbirth in rare cases
**Varicella zoster virus**	*α-herpesviridae*	Humans	Aerosols, vesicles	Miscarriage, FGR, congenital varicella syndromes(skin and limb malformation, cataracts, microcephaly, hydrocephalus etc.)
**Cytomegalovirus**	*β-herpesviridae*	Humans, monkeys	Direct contact (bodily fluids, blood, saliva, urine and breastmilk)	Premature birth, FGR, congenital disorders (microcephaly, hearing loss, vision loss, seisure, intellectual disability etc.)
**Coxsackievirus B**	*Picornaviridae*	Humans	Aerosols, fecal-oral route	Miscarriage, stillbirth, fetal sepsis
**Zika virus**	*Flaviviridae*	Humans, monkeys	Mosquito, sexual	Miscarriage, microcephaly
**Dengue virus**	*Flaviviridae*	Humans	Mosquito, breast milk	Miscarriage, premature birth, stillbirth
**Ebola virus**	*Filoviridae*	Humans, bats, primates	Blood, bodily fluids	Miscarriage, stillbirth
**Marburg virus**	*Filoviridae*	Human, bats	Blood, bodily fluids	Miscarriage, stillbirth
**Human immunodeficiency virus**	*Retroviridae*	Humans	Blood, bodily fluids	Miscarriage, stillbirth
**Parvovirus B19**	*Parvoviridae*	Humans	Aerosols, saliva, blood	Miscarriage, fetal anemia, nonimmune hydrops fetalis
**Lassa virus**	*Arenaviridae*	Humans, rodents	Aerosols, contact with infected rodent hosts	Perinatal mortality

**Table 2:** Comparison of commonly used animal models used to study human placentation.

Animal	Similarities	Differences	References
**Mice**	Chorioallantoic placenta Discoid, hemochorial placenta	Gross morphology and specific trophoblast cell types Yolk sac as major player in maternal–fetal exchange Placental labyrinth Trophoblast invasion limited to decidua Maternal and fetal blood separated by three trophoblast layers	[100,101,102]
**Guinea pigs**	Trophoblast invasion Hemochorial interface Pattern of placental development, especially the distribution pattern of trophoblast cell proliferation	Lobulated placenta with lobes as circulatory units Labyrinthine placentation	[103,104,105]
**Horses**	Trophoblast population has human counterparts with conserved essential properties Conserved transcription factors for trophoblast differentiation Extended length of gestation	Diffuse placenta with maternal–fetal exchange occurring across all available surfaces Temporal and spatial MHC expression regulation Endometrial epithelium, connective tissue, and uterine endothelium present	[86,106,107,108]
**Non-human primates**	Long gestational period Discoid, hemochorial placenta	Rapid but shallow trophoblast invasion	[109,110,111]
